# LigaSure Haemorrhoidectomy versus Conventional Diathermy for IV-Degree Haemorrhoids: Is It the Treatment of Choice? A Randomized, Clinical Trial

**DOI:** 10.5402/2011/467258

**Published:** 2010-11-21

**Authors:** Maurizio Gentile, Michele De Rosa, Gabriele Carbone, Vincenzo Pilone, Francesca Mosella, Pietro Forestieri

**Affiliations:** Department of General, Oncological, and Videoassisted Surgery, University of Naples “Federico II”, 80131 Naples, Italy

## Abstract

*Introduction*. Milligan-Morgan haemorrhoidectomy performed with LigaSure system (LS) seems to be mainly effective where a large tissue demolition is required. This randomized study is designed to compare LigaSure haemorrohidectomy with conventional diathermy (CD) for treatment of IV-degree haemorrhoids. *Methods*. 52 patients with IV-degree haemorrhoids were randomized to two groups (conventional diathermy versus LigaSure haemorrhoidectomy). They were evaluated on the basis of the following main outcomes: mean operative time, postoperative pain, day of discharge, early and late complications. The time of recovery of work was also assessed. All patients had a minimum follow-up of twelve months (range 12–24). All data were statistically evaluated. *Results*. 27 patients were treated by conventional diathermy, 25 by LigaSure. The mean operative time was significantly shorter in LS, such as postoperative pain, mainly lower on the third and fourth postoperative day: moreover pain disappeared earlier in LS than CD. The time off-work was shorter in LS, while there was no difference in hospital stay and overall complications rate. *Conclusions*. LigaSure is an effective instrument when a large tissue demolition is required. This study supports its use as treatment of choice for IV degree haemorrhoids, even if the procedure is more expansive than conventional operation.

## 1. Introduction

Surgery is actually indicated for patients with grade III and IV haemorrhoids. The traditional Milligan-Morgan operation and the Ferguson one in United States are still the most used and effective approaches for patients with symptomatic haemorrhoids of III and IV degrees [1–3]. These two procedures have similar possible complications, such as blood loss and postoperative pain, which can cause a prolonged hospital stay: this can be considered as a “social” problem, since a fast wound healing would allow a quicker return to work habits and daily activities [4]. Several papers looking for the optimal treatment of haemorrhoids have been published in recent years and new devices and procedures have been proposed to overcome haemorrhoidectomy complications: such tools as stapling haemorrhoidopexy and Doppler-guided haemorrhoidal vessel ligation are based on principles conceptually different from excisional surgery [5].

The LigaSure Vessel Sealing System has been recently introduced [6] as an instrument conceived to upgrade the conventional treatment of haemorrhoids: it consists of a bipolar electrothermal device which offers an optimised combination of pressure and radiofrequency, sealing blood vessels up to 7 mm in diameter and generating an energy tailored to the tissue impedance, with a thermal injury confined to 2 mm over the surgical site. This limited spread reduces anal spasm and allows to perform a bloodless haemorrhoidectomy with reduced postoperative pain and fast healing. Thus this operation can be recommended as the ideal technique because of the potential reduction in tissue trauma [7].

The main goal of some randomized trials was to evaluate the benefits of the system over traditional approaches [8–10]: although an overall favourable trend exists toward LigaSure, conclusions are not univocal and definitive; this creates some uncertainty, also considering the increasing cost for the use of the disposable device: thus it is essential to keep on experimenting to determine whenever a true advantage exists [11, 12].

While it is still debated which is the “gold standard” for III-degree, there is a large agreement that the traditional Milligan-Morgan operation (and the Ferguson in United States) is the most effective treatment for IV-degree haemorrhoids [2]: as reported by Ortiz, stapled haemorrhoidopexy was not effective as a definitive cure for the symptoms of prolapse and itching in patients with fourth-degree haemorrhoids [13].

Thus conventional diathermy haemorrhoidectomy should continue to be recommended in patients with symptomatic, prolapsed, and irreducible piles.

According to the belief that a conventional haemorrhoidectomy is the only effective treatment in IV-degree patients, this prospective study was designed to verify if the use of the LigaSure system can be proposed as a less painful and bloodless alternative where a large tissue demolition is required.

## 2. Methods

Between June 2007 and June 2008, 52 patients underwent surgical treatment for IV-degree haemorrhoids with two different techniques (conventional diathermy versus LigaSure haemorrhoidectomy). They were part of the whole group of 128 patients operated in the same period in our department for haemorrhoids (III-IV degree).

Inclusion criteria were bleeding and permanently prolapsing haemorrhoids in IV grade according to Nivatvongs [14], age range 19–80 years, ASA I-II, both genders. Patients with previous or concomitant anorectal diseases were excluded such as those with permanently prolapsed haemorrhoids limited to one quadrant only.

All patients were evaluated preoperatively with a complete proctological examination including past proctologic history, continence evaluation (Wexner continence score) [15], and anoscopy: a colonoscopy was performed in those aging over 50 years to rule out colonic cancer. 

The project of the study was submitted to obtain the University Ethical Commission approval.

After a complete and comprehensive explanation from a member of the surgical team, an informed consent was subscribed by patients.

Patients enrolled for the study were divided into two groups by using a computer-generated list for randomization: the code enclosed in a numbered envelope corresponding to one of the two techniques was shown at the beginning of the operation to the surgeon.

All patients were operated by the same senior staff surgeon (MG) as day-surgery procedures under epidural or general anaesthesia.

All patients were required to record pain from the first postoperative day until the 28th postoperative day on a self-administered VAS scale in cm (0–10): it was required to record an overall pain score of the day either at defecation of at rest (about twelve hours after defecation).

Postoperative complications were defined as “immediate” within the first month after surgery and “late” after the first month.

Patients were assessed one week, one month, six, and twelve months after the operation. Anything concerning operative time, postoperative pain, day of discharge, early and late complications was recorded. Time to return to work was also assessed. All data were collected by an independent observer not from the surgical team and the assessed outcome was not blinded.

### 2.1. Operative Technique

As a preoperative protocol, both groups of patients were cleaned by a saline enema on the evening before the operation and 500 mg of metronidazole were given intravenously at the beginning of the procedure and continued for the whole following week.

Patients received analgesic administration of tramadol in continuous infusion by elastomeric pump for about twelve hours as PCA (patient controlled analgesia) and, after hospital discharge, analgesia was achieved by 10 mg ketorolac on demand (never more than three times a day).

The operation was performed in lithotomy position in both groups.

In the LigaSure group the procedure was carried out by using a Fansler retractor and performed by applying LigaSure forceps to the level of the vascular pedicle: scissors were then used to cut along the line of coagulum, lifting the pile from the internal sphincter. The vascular pedicle was sealed by LigaSure without transfiction. The wound was left open. The procedure was repeated for each quadrant. A haemostatic sponge was left in the anal canal after an accurate inspection of the area by an Eisenhammer retractor.

In diathermy group, a conventional haemorrhoidectomy was performed according to the technique described by Loder and Phillips [16]. The piles were lifted from the internal sphincter by diathermy, and the vascular pedicle was sutured. The wounds were left open. An anal sponge was left into the anal canal at the end of the procedure after inspection by an Eisenhammer retractor.

### 2.2. Statistical Analysis

Data were expressed as median values, and values of less than  .05 were considered statistically significant. Mann-Whitney *U*-test was used for postoperative pain between Diathermy and LigaSure groups. Fisher exact test was used for the incidence of postoperative complications.

The study power was considered statistically significant with a reduction of postoperative anal pain of 20% at least with an alpha error of 5% and beta error of 10%. At least 22 patients were needed for each arm of the study according to Pocock's formula.

## 3. Results

52 patients with IV-degree haemorrhoids were randomly divided into two groups: 27 patients underwent conventional Milligan-Morgan operation and 25 were treated by LigaSure haemorrhoidectomy. All patients had a minimum followup of twelve months (range 12–24).

The two groups were comparable for age (mean age: 47 for diathermy, 48 for LigaSure patients; overall range 19–80), gender (male/female ratio not statistically significant), working activities and symptoms (Table 1).

No patient was preoperatively incontinent (Wexner continence score: 0–4).

No patient was lost at the followup.

**Figure 1:**
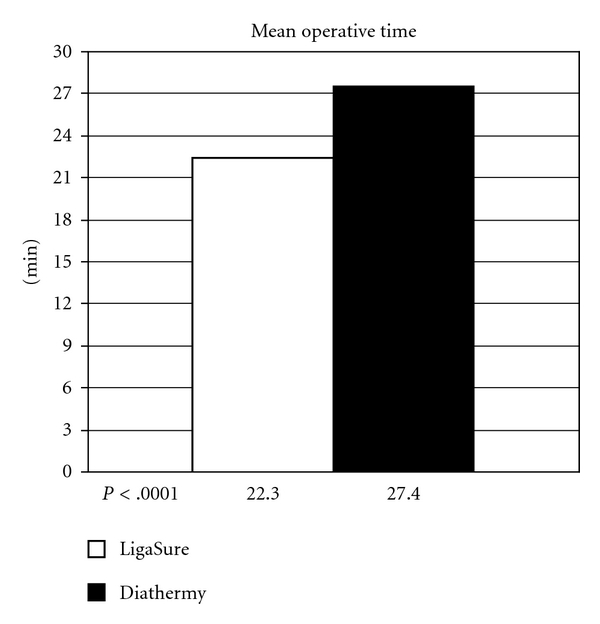
Mean operative time in the two groups. LigaSure operation is significantly faster.

The mean operative time for the LigaSure group was 22.3 minutes compared to 27.4 for conventional diathermy, with a statistically significant difference (*P* < .0001) (Figure 1).

There was no difference in hospital stay since patients were discharged 24 ± 2 hours after the operation in both groups, and delayed discharges were registered in two cases of each group (III postoperative day) due to minor bleeding (2 conventional versus 1 LigaSure) and acute urinary retention (1 LigaSure) (*P* = 1 NS).

The overall incidence of complications was not different between the two groups: 9 patients (33%) after conventional diathermy versus 7 (28%) in LigaSure group (*P* = .7683 NS).

Among early postoperative complications, three patients in conventional diathermy group had minor bleeding compared to one in the LigaSure group (*P* = .6110 NS), but none of these patients required reintervention.

Three patients were observed for two days after operation and discharged on third postoperative day; another patient required a package of haemostatic absorbable sponge. Finally one anal fissure was observed in the diathermy group at one month followup (*P* = 1).

As late complications, one anal stenosis was detected in the LigaSure group at six-month followup (*P* = .4808 NS): it was treated by anal dilator associated with a nifedipine topic ointment with a good final result. Moreover, an incomplete healing was observed in three patients (11.1%) of the diathermy group compared to two patients (8%) of the LigaSure one: the difference was not statistically significant (*P* = .6624) (Table 2).

**Figure 2:**
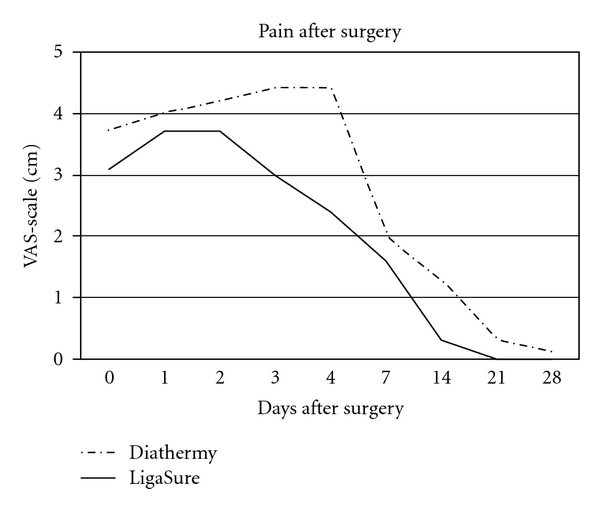
Pain after surgery in the two groups. Incidence is significantly different 3 and 4 days after operation.

Postoperative pain was well controlled after the operation by continuous infusion in both groups: due to this effective administration, the difference was not significant after the first 12 hours in both groups (*P* = .0799 NS) and during the first postoperative day (3.7 VAS score versus 4.0 VAS score *P* = .0408 NS), while a statistically significant difference was observed three (3.14 versus 4.46 VAS score, *P* < .0002) and four days after the operation (2.43 versus 4.42 VAS score, *P* < .0001) with a lower need of analgesic drugs in the LigaSure group: after one week, the decrease of the pain was similar in the two groups (1.6 versus 2.0 VAS score, *P* = .2356 NS) but the LigaSure patients were closer to the baseline (no pain at all) at the 14th day (0.3 versus 1.3 VAS score, *P* < .0042) earlier than conventional diathermy patients. Finally, 21 and 28 days after operation, both values were not significant (0 versus 0.3 *P* = .37722 VAS score at 21th day NS) (Figure 2).

**Figure 3:**
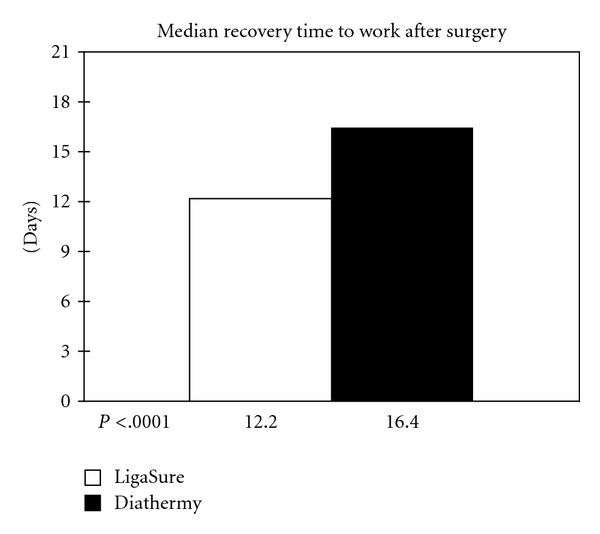
Median recovery time after surgery. Patients have a faster return to work after LigaSure.

Patients treated by LigaSure haemorrhoidectomy returned to work activities 12.2 days after the operation compared to 16.4 days of conventional diathermy group and this is a strongly significant difference toward LigaSure system (*P* < .0001) (Figure 3).

## 4. Discussion

Conventional diathermy haemorrhoidectomy is still recommended in patients with symptomatic, prolapsed, irreducible piles.

The LigaSure vessel sealing system is one of the tools recently introduced to overcome haemorrhoidectomy's major complaints and has been compared to conventional diathermy and Ferguson's closed haemorrhoidectomy in several published randomized trials [8–11]. It is difficult to achieve an univocal evidence of benefits from its use in terms of postoperative pain and analgesic requirements: unfortunately, these studies enrolled a small number of patients and the evaluation of subjective symptoms is uncertain.

In a large meta-analysis of eleven trials and 1046 patient, Mastakov et al. [17] confirmed the evidence that LigaSure haemorrhoidectomy is effective: almost all outcome parameters are better in the radiofrequency group except for the overall incidence of complications reported that was not significant. Altomare and the Italian LigaSure Study Group [18] in a prospective multicentric randomized trial on 273 patients showed a significant reduction in postoperative pain, a shorter operating time, and a faster return to work, but no difference in the incidence of postoperative bleeding and late complications up to 28 days after operation.

Finally Milito et al. [19] in a review of eleven randomized trials with a total of 850 patients reported a significant improvement in postoperative pain, wound healing, and time off work, but no difference in postoperative bleeding and complications between the two groups was found.

Kraemer et al. [20] compared LigaSure with stapled haemorrhoidopexy, which has a better reputation for postoperative pain, with a slightly favourable trend of radiofrequency in the outcome of patients with fourth-degree piles.

However, all these experiences reported data about the overall groups of III-and IV-degree haemorrhoids patients.

In a different way, the present study compares the LigaSure system to conventional diathermy, merely considering a group of IV-degree patients, where the Milligan-Morgan is the treatment of choice.

The LigaSure is effective: according to most studies the use of this device allows a shorter operating time with a statistically significant difference (22.3 versus 27.4 min). Moreover, the system is simple and easy to learn and the mean time reported in our series, ranging from 17.2 to 27.4 minutes, is mainly due to the size of the piles than to the “learning curve”.

A reduction in postoperative pain score, due to a minor tissue damage, and a faster wound healing are reported: the trend does not differ between the two groups early after the operation and in the first postoperative day, but it becomes significant during the third and fourth postoperative days, decreasing similarly in the two groups one week after surgery.

Finally patients with LigaSure haemorrhoidectomy are free from pain earlier than those with conventional diathermy.

A similar incidence of early postoperative pain in both groups can be explained by the use of analgesic infusion by elastomeric pump during the first twelve hours after operation, while the trend is different when patients received pain-killer pills on demand.

In our experience, patients recorded the pain with an overall score for each day: differently Altomare et al. [18] considered the pain either after evacuation or at rest (12 hours after) with a significant difference between the two groups only in the pain considered at rest; otherwise, the evacuation, with its mechanical stimulation of the anoderm, produces the same pain with any device used.

Moreover, the absence of sutures transfixing vascular pedicles could be another additional advantage in reducing pain: it avoids the development of local ischemia and necrosis that might cause acute postoperative pain and secondary bleeding [19].

LigaSure haemorrhoidectomy is safe: in our experience, a low rate of postoperative complications was detected and the overall incidence does not significantly differ between the two groups (*P* = .7683 NS), even with a follow-up prolonged up to 24 months.

All patients were managed as day-surgery cases, and a delayed discharge (III postoperative day) was recorded only in two cases for each group (1 bleeding and 1 acute urinary retention in the LigaSure group and 2 blood loss in conventional diathermy).

These patients were managed conservatively and there was no need to redo surgery.

Recent reports suggest that LigaSure cannot be considered a method without complications and it has been described that thermal injury can contribute to develop an anal stenosis: Filingeri et al. [21] report four cases of stenosis out of 203 LigaSure procedures (2%), one out 42 patients in Wang's series [22].

In our experience of IV-degree patients (higher risk), only one case was detected and the incidence seems to be in line with the data of the literature (4-5%) [17, 19].

This complication was treated by anal dilators and nifedipine topic ointment with a good final result: however, as a useful trick to avoid the development of a circular scar, we always preserve intact anoderm and mucosal bridges between the wounds. A quicker healing and a more comfortable condition support also a faster return to daily activities: patients of the LigaSure group returned to work activities in a significantly shorter time than conventional diathermy patients (12.2 versus 16.4 days, *P* < .0001). Chung and Wu [23] found no difference in this outcome parameter while Milito et al. reported a shorter time off-work after LigaSure haemorrhoidectomy compared to other techniques (*P* < .001), and Sayfan et al. [6] observed a shorter convalescence period (7.4 versus 18.6 days). Finally Altomare et al. pointed out that the difference is particularly evident in in those patients who do not draw any possible advantages from longer convalescence time, while there is only a moderate trend in patients in the workforce [18].

Regarding LigaSure safety, no recurrence was detected as well as no complaint for any kind of incontinence due to sphincter damage: same results were recorded in conventional diathermy group. Despite the fact that our patients had a short followup, results from large clinical trials confirmed the benefits of the technique in a mid-term perspective if the device was correctly applied.

Finally, in our experience LigaSure haemorrhoidectomy resulted in a shorter operative time and a lower postoperative pain, with a faster return to work and an overall complications rate similar to conventional diathermy: moreover, the procedure is safe, with a minimum risk of impaired continence.

As far as economic considerations are concerned, the additional cost of the disposable device (approximately 230 euros) is balanced by a shorter operative time, the possibility of a day-case surgery, and an earlier return to work.

## 5. Conclusions

This prospective controlled randomized trial confirms the advantages of LigaSure haemorrhoidectomy over conventional diathermy when a large tissue demolition is required supporting the use of this device as the treatment of choice in IV-degree haemorrhoids. Its additional cost to each procedure finally results in a significant cost-saving.

A limitation of the present study can be identified in the small size of the sample and the limited followup: thus, the benefits of LigaSure as a low-pain and long-term effective technique need to be further evaluated in larger series.

## Figures and Tables

**Table 1:** Patients characteristics in the two groups.

	Diathermy (25 pts)	LigaSure (27 pts)	*P* value
Age (years) (range)	49.4 (18–75)	47.2 (18–75)	NS
Male/Female ratio	1.4	1.3	NS
Wexner Continence score (range)	0 (0–2)	0 (0–2)	NS
Bleeding (pts) (%)	24 (96%)	25 (92.5%)	*P* = 1
Pain (pts) (%)	18 (72%)	21 (77.7%)	*P* = .7523
Itching (pts) (%)	17 (68%)	23 (85.1%)	*P* = .1933

**Table 2:** Complications after surgery in the two groups.

	Diathermy (pts) (%)	LigaSure (pts) (%)	*P* value
Delayed discharge	2	2	*P* = 1
Minor bleeding	3	1	*P* = .6110
Acute urinary retention	0	1	*P* = 1
Anal fissure	1	0	*P* = 1
Incomplete healing	3	2	*P* = .6624
Anal stenosis	0	1	*P* = .4808
Sphincter damage	0	0	*P* = 1
Overall complication rate	9 (33.3%)	7 (28%)	*P* = .7683
